# Supplementary material for: The interactome of intact mitochondria by cross-linking mass spectrometry provides evidence for coexisting respiratory supercomplexes
Source: Mol Cell Proteomics. 2017 Dec 8;17(2):216–32. doi: 10.1074/mcp.RA117.000470 (PMC5795388; doi:10.1074/mcp.RA117.000470)
Supplement: Supplemental Data [file supp_RA117.000470_133922_0_supp_23978_fzffwf.zip › spectra_annotation/mito_DR_spectra_annotation/233-2-9-1-14-1.pdf]

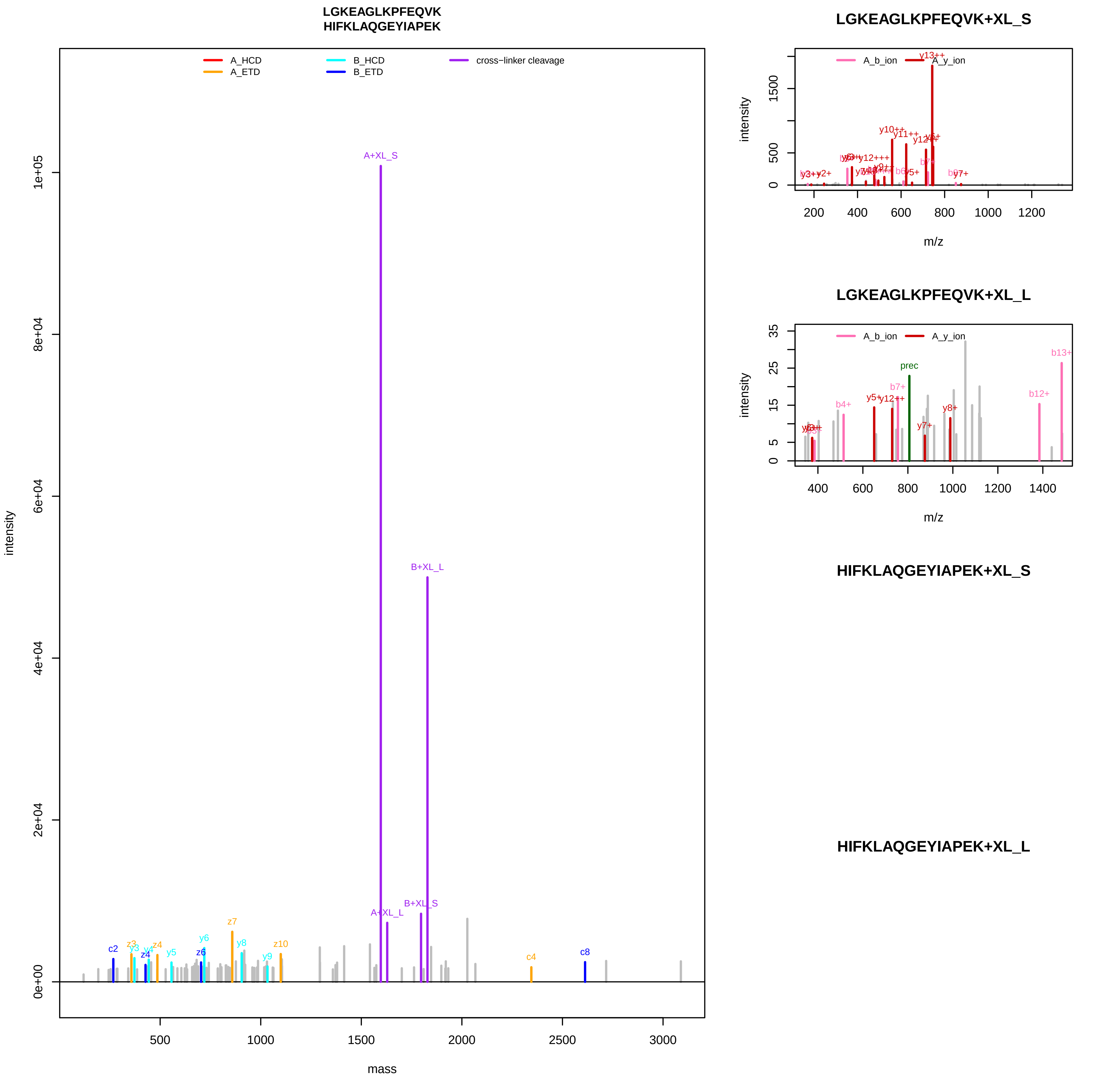

LGKEAGLKPFEQVK
HIFKLAQGEYIAPEK
A_HCD
A_ETD
B_HCD
B_ETD
cross−linker cleavage
A+XL_S
B+XL_L
A+XL_L
B+XL_S
c2
z3
y3
z4
y4
z4
y5
z6
y6
z7
y8
y9
z10
c4
c8
intensity
mass
0e+00
2e+04
4e+04
6e+04
8e+04
1e+05
500
1000
1500
2000
2500
3000
LGKEAGLKPFEQVK+XL_S
A_b_ion
A_y_ion
y13++
y10++
y11++
y12++
y12+++
y5+
y7+
y3+
y2+
intensity
m/z
0
500
1500
200
400
600
800
1000
1200
LGKEAGLKPFEQVK+XL_L
A_b_ion
A_y_ion
prec
b4+
b7+
y5+
y12++
y7+
y8+
b12+
b13+
intensity
m/z
0
5
15
25
35
400
600
800
1000
1200
1400
HIFKLAQGEYIAPEK+XL_S
HIFKLAQGEYIAPEK+XL_L
